# Counting Degrons: Lessons From Multivalent Substrates for Targeted Protein Degradation

**DOI:** 10.3389/fphys.2022.913063

**Published:** 2022-07-04

**Authors:** Cynthia N. Okoye, Pamela J. E. Rowling, Laura S. Itzhaki, Catherine Lindon

**Affiliations:** Department of Pharmacology, University of Cambridge, Cambridge, United Kingdom

**Keywords:** targeted protein degradation, ubiquitin ligase, degron, SLiM, E3-substrate interaction, multivalency

## Abstract

E3s comprise a structurally diverse group of at least 800 members, most of which target multiple substrates through specific and regulated protein-protein interactions. These interactions typically rely on short linear motifs (SLiMs), called “degrons”, in an intrinsically disordered region (IDR) of the substrate, with variable rules of engagement governing different E3-docking events. These rules of engagement are of importance to the field of targeted protein degradation (TPD), where substrate ubiquitination and destruction require tools to effectively harness ubiquitin ligases (E3s). Substrates are often found to contain multiple degrons, or multiple copies of a degron, contributing to the affinity and selectivity of the substrate for its E3. One important paradigm for E3-substrate docking is presented by the Anaphase-Promoting Complex/Cyclosome (APC/C), a multi-subunit E3 ligase that targets hundreds of proteins for destruction during mitotic exit. APC/C substrate targeting takes place in an ordered manner thought to depend on tightly regulated interactions of substrates, with docking sites provided by the substoichiometric APC/C substrate adaptors and coactivators, Cdc20 or Cdh1/FZR1. Both structural and functional studies of individual APC/C substrates indicate that productive ubiquitination usually requires more than one degron, and that degrons are of different types docking to distinct sites on the coactivators. However, the dynamic nature of APC/C substrate recruitment, and the influence of multiple degrons, remains poorly understood. Here we review the significance of multiple degrons in a number of E3-substrate interactions that have been studied in detail, illustrating distinct kinetic effects of multivalency and allovalency, before addressing the role of multiple degrons in APC/C substrates, key to understanding ordered substrate destruction by APC/C. Lastly, we consider how lessons learnt from these studies can be applied in the design of TPD tools.

## 1 Introduction

Interactions between E3 ligases and their substrates occur through interaction with “degrons”—short linear motifs (SLiMs) within intrinsically disordered regions of substrates, which are typically of low affinity (Min et al., 2013; Guharoy et al., 2016). In many examples explored to date, target discrimination and productive complex formation (leading to degradation of substrate) rely on interaction through multiple degrons (Karamysheva et al., 2009; Di Fiore et al., 2015; Pierce et al., 2016; Tian et al., 2018). Degradation of a substrate, in general, requires extensive sequential ubiquitination of the substrate through multiple rounds of recruitment of a ubiquitin-loaded conjugating (E2) enzyme to the E3-substrate complex, so there is a clear relationship between E3-substrate affinity and rate and/or timing of substrate degradation. Although the presence of multiple degrons is frequently invoked as a critical element in the targeting of substrates by their E3s, there is no single explanation governing the kinetic implications of such a feature. An intuitive explanation is the increased affinity and specificity afforded by avidity effects (by increasing the frequency of productive collisions and/or by increasing the interaction surface thus slowing down dissociation since multiple interactions must break), but additionally cooperative binding at different degron receptor sites on the E3 can also occur (Hartooni et al., 2022). Avidity depends upon multimerization of the E3 (as has been described for SPOP and Keap1) (McMahon et al., 2006; Canning et al., 2015; Marzahn et al., 2016) or on the presence of multiple degron receptor sites on a monomeric E3 (as exemplified by APC/C) (He et al., 2013). However, for at least one example of a substrate studied in great detail, Sic1, the presence of repeated degrons is proposed to favour the kinetics of interaction at a single site on its E3, a phenomenon known as allovalency (Kõivomägi et al., 2011).

**FIGURE 1:**
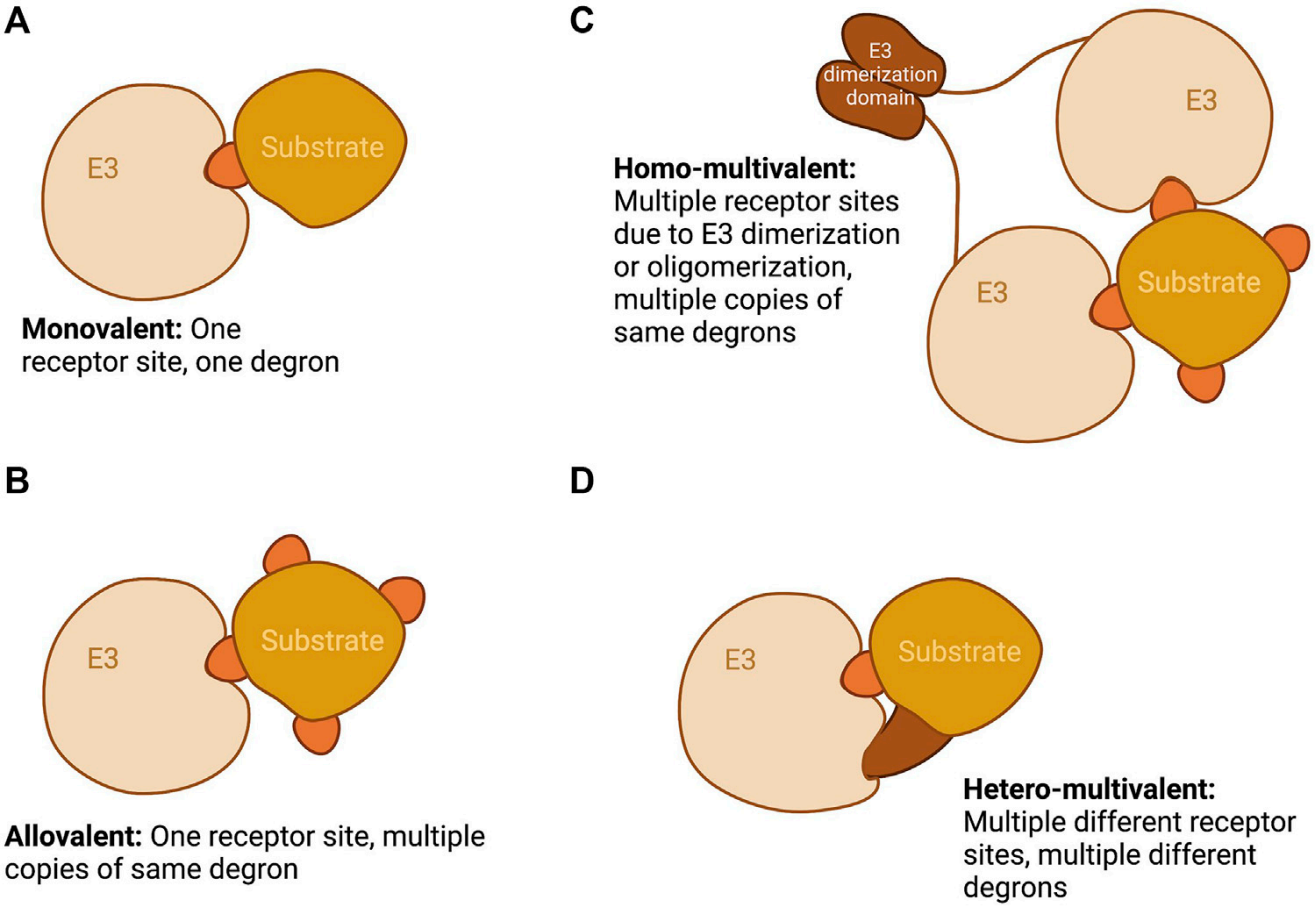
Schematic showing the different modalities of E3-substrate recognition namely: **(A)** Monovalent, one E3 receptor site and one degron; **(B)** Allovalent, one E3 receptor site and multiple copies of the same degron; **(C)** Homo-multivalent, multiple E3 receptor sites as a result of E3 dimerization or oligomerization and multiple copies of the same degron; and **(D)** Hetero-multivalent, multiple different receptor sites on an E3 subunit and multiple different degrons. Created with BioRender.com.

Recent reviews on SLiM-mediated interactions have discussed in detail the kinetic implications of multivalency (Olsen et al., 2017; Ivarsson and Jemth, 2019). Here we describe examples of substrate-E3 interactions that illustrate different modes of multivalent binding (illustrated in Figure 1) before reviewing what is known about the control of APC/C binding to its substrates. We consider whether the multiple degrons in APC/C substrates represent alternative or composite docking sites and discuss evidence that multivalency plays a role in substrate ordering in mitosis.

## 2 The Mechanisms of Multi-Degron Recognition

### 2.1 Co-Operative Binding

#### 2.1.1 Nrf2/Keap1

A major pathway of cellular protection from oxidative and electrophilic damage involves the basic leucine zipper (bZip) transcription factor, Nrf2, and the Cullin Ring Ligase (CRL) Keap1-CUL3-RBX1 where Keap1 serves as the substrate recognition subunit (Ma, 2013). Key domains in Keap1 are the BTB domain that binds CUL3 and mediates homodimerization of the E3, and a KELCH domain that contains the receptor site for degron binding (Furukawa and Xiong, 2005). Under normal conditions, low levels of Nrf2 are maintained through its ubiquitination by Keap1-CUL3-RBX1 and subsequent degradation by the proteasome. However, in conditions of oxidative stress, Keap1 undergoes oxidation of its cysteine residues, and this leads to conformational changes that result in the dissociation of Nrf2 from Keap1 and subsequent Nrf2 stabilization (Rachakonda et al., 2008; Kobayashi et al., 2009; Sekhar et al., 2010). Nrf2 then heterodimerizes with Maf proteins to increase the expression of genes under the regulation of antioxidant responsive elements (ARE) (Chan and Kwong, 2000; McMahon et al., 2001; Katsuoka et al., 2005; Surh et al., 2008).

**FIGURE 2:**
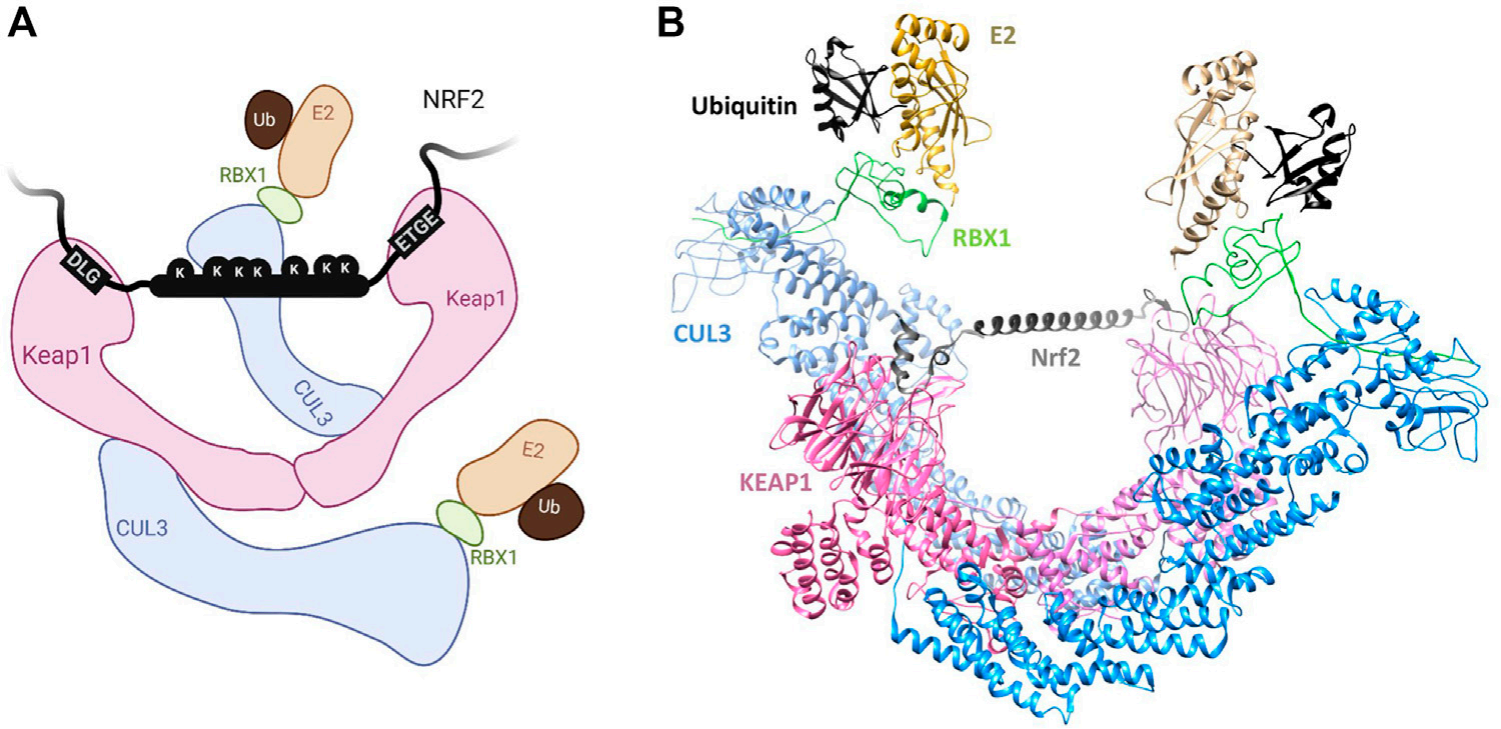
**(A)** Schematic of the Keap1-Nrf2 interaction described in Section 2.1.1. Created with BioRender.com. **(B)** Structural model of the full Keap1-CUL3-RBX1 complex bound to Nrf2 assembled as described previously (structural model kindly provided by A. Bullock, University of Oxford) (Canning et al., 2013). A predicted helix in the Neh2 region of Nrf2 is modelled between the bound DLG and ETGE sites.

In the intrinsically disordered Nrf2-ECH homology 2 (Neh2) domain of Nrf2 are two degrons, with sequences ETGE and DLG, through which Nrf2 binds Keap1 (Tong Kit I. et al., 2006). Each degron binds to the same receptor site on the dimeric Keap1 in a homo-multivalent fashion (Figure 1C). Although short peptides containing either degron alone can bind Keap1 (Canning et al., 2015), the DLG degron binds with approximately two orders of magnitude more weakly than does ETGE (Tong Kit I. et al., 2006). Nonetheless, the DLG motif is required for efficient ubiquitination and degradation of Nrf2 (Tong et al., 2007). Additional electrostatic interactions of the ETGE residues result in increased favorable enthalpic contributions to its stronger binding affinity. As for kinetics, binding of the ETGE motif follows slow association and dissociation rates, whereas that of DLG follows fast association and dissociation rates (Fukutomi et al., 2014; Canning et al., 2015). DLG binding is therefore dependent on ETGE docking in cooperative fashion. The Nrf2/Keap1 binding mechanism is often described as “hinge-and-latch”, whereby the higher affinity ETGE acts as the hinge and the lower affinity DLG as the latch (Tong et al., 2007), as can be further rationalised by models of the full-length Keap1 dimer (Figure 2). Nrf2 spans the gap between the two KELCH domains of the Keap1 dimer, which each engage the ETGE and DLG motifs. The region between the two degrons is predicted to have a helical structure and contains seven lysine residues that may be ubiquitination sites, suggesting that the dimeric Keap1 structure is required to position this Nrf2 segment for efficient ubiquitination (McMahon et al., 2006; Tong et al., 2007; Canning et al., 2015).

#### 2.1.2 Gli3/SPOP

Another prominent CRL that utilizes the multi-degron recognition paradigm through E3 multimerization (Figure 1C) is SPOP-CUL3-RBX1. SPOP is the substrate recognition subunit in this E3 ligase complex, and contains an N-terminal MATH domain, a BTB domain and a C-terminal BACK domain. Substrates of SPOP often contain multiple SPOP-binding consensus motifs (SBMs)—nonpolar-polar-S-S/T-S/T—that bind to the SPOP MATH domain (Zhang et al., 2009; Zhuang et al., 2009), while E3 dimerization and oligomerization occur through the BTB and BACK domains (van Geersdaele et al., 2013; Marzahn et al., 2016). Both the oligomerization of the E3 and the binding of substrates are implicated in liquid-liquid phase separation (LLPS) of the E3-substrate complexes, which serves to effectively increase local concentrations and create ubiquitination hotspots (Marzahn et al., 2016). This LLPS property is important for efficient ubiquitination of many SPOP substrates but not all of them (Usher et al., 2021). For example, one important target of SPOP is MyD88 which is a signal transducer involved in NF-κB signalling. MyD88 has been found to contain one SPOP-binding motif (SBM) within its intermediate (INT) domain that is essential for its binding to SPOP and subsequent ubiquitination and degradation (Guillamot et al., 2019; Li et al., 2020). On the other hand, the MATH domain of SPOP is required for its interaction with MyD88 whereas the BTB and BACK domains are less critical (Li et al., 2020). Therefore, the MyD88-SPOP interaction is likely of a monovalent fashion (Figure 1A) and unlikely to be dependent on LLPS.

**FIGURE 3:**
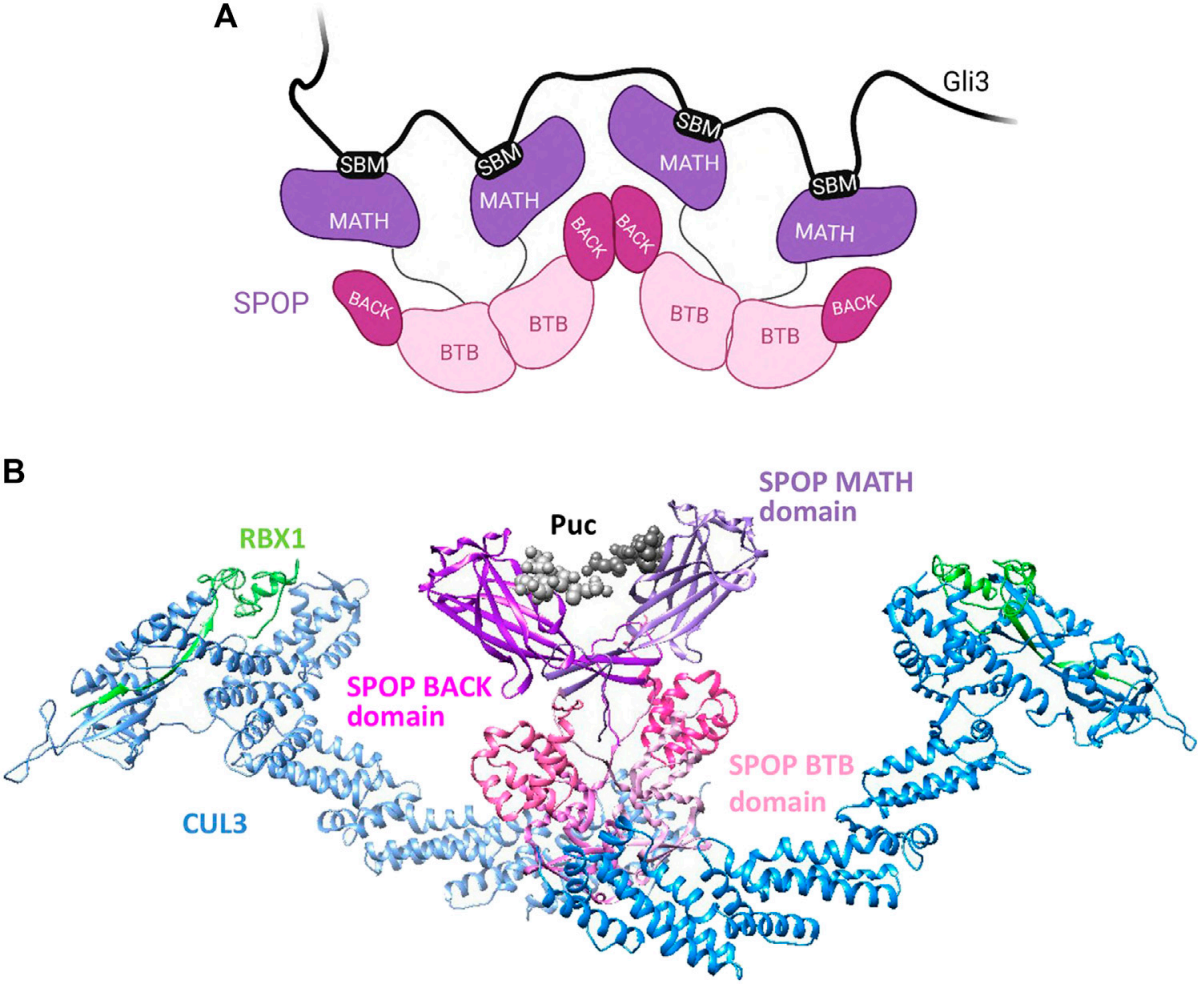
**(A)** Schematic showing the oligomerization of SPOP via its BACK and BTB domains, described in Section 2.1.2. Created with BioRender.com. **(B)** Model of SPOP dimer bound to a degron of the substrate Puc. The proteins are coloured: Cul3, blue; Rbx1, green, and Puc in black. SPOP domains are coloured: MATH degron-binding domain in purple; BTB domain in pink, and BACK domain in magenta. The model was made in Chimera (https://www.rbvi.ucsf.edu/chimera) using the following PDBs, 3IVV, 4EOZ, 3HQI and 1LDJ. (Zheng et al., 2002; Zhuang et al., 2009; Ji and Privé, 2013).

SPOP also plays a vital role in the regulation of Hedgehog (Hh) signaling by targeting Gli2/3 for degradation (Wen et al., 2010; Umberger and Ogden, 2021). The Hh signaling pathway is crucial for proper embryonic development, maintenance of stem cells, and repair of tissues, and abberancies in Hh signaling have been implicated in cancers (Skoda et al., 2018). A key player in the Hh pathway is Gli3, a transcriptional regulator of the pathway and a well-studied SPOP substrate that contains as many as ten SBMs (Zhang et al., 2009). Biophysical studies with truncated Gli3 N-terminal fragments reveal that each SBM has rather weak affinity for SPOP in the millimolar to micromolar range, and that as many as three SPOP^MATH^ molecules can bind one molecule of the N-terminal Gli3 fragment (Pierce et al., 2016). Additionally, compared to monomeric SPOP, oligomeric SPOP not only binds the Gli3 fragment with increased affinity but also more efficiently ubiquitinates the fragment *in vitro*. All SBMs in Gli3 are important for binding and/or ubiquitination and possibly also for LLPS (Pierce et al., 2016). It has been hypothesised that the phase separation of SPOP-substrate complexes is not solely due to intrinsic SPOP oligomerization but can also be driven by substrates that contain multiple SBMs. For example, Daxx is a multivalent SPOP substrate that contains at least eight SBMs and undergoes LLPS with SPOP, but a Daxx mutant with lower multivalency displays reduced LLPS and is also less ubiquitinated. Another substrate Pdx1, found to contain only two SBMs, does not phase separate with SPOP (Usher et al., 2021). Therefore, the presence of many SBMs in SPOP substrates can serve as a driver of high-affinity binding as well as phase separation with SPOP. The ability of SPOP to dimerize and oligomerize (Figure 3) allows for the utilization of multiple valency modalities, namely monovalency (with MyD88) and homo-multivalency (with Gli3), to effectively target a diverse array of substrates.

#### 2.1.3 Cyclin E/FBXW7

Cyclin E-CDK2 is an important regulator of the cell cycle that controls the G1/S transition. Cyclin E (CCNE1) turnover is mainly regulated by SCF^FBXW7^, the SKP1-CUL1-F-box (SCF) ubiquitin ligase complex containing FBXW7. Like other F-box targets, CCNE1 contains so-called two “phospho-degrons” that are phosphorylated at residues T62 and T380/S384 by CDK2, GSK3 and other kinases (Welcker et al., 2003). The pT380/pS384 phosphodegron is the highest affinity degron in CCNE1 and is sufficient for substrate turnover, whereas pT62 is of lower affinity but is important for phosphorylation of T380 (Ye et al., 2004; Hao et al., 2007; Welcker et al., 2013). FBXW7 can form dimers via its dimerization domain, and both the pT62 and pT380/pS384 phospho-degrons can simultaneously bind dimerized FBXW7. However, dimerization of FBXW7 is more crucial for suboptimal (low-affinity) degrons, and since CCNE1 contains a robust (high-affinity) degron, its turnover is not contingent on FBXW7 dimerization (Hao et al., 2007; Welcker et al., 2013) (Figure 1A). On the other hand, SREBP1 is an example of a substrate that contains a suboptimal degron and is highly dependent on FBXW7 dimerization for its turnover (Sundqvist et al., 2005; Bengoechea-Alonso and Ericsson, 2009; Welcker et al., 2013) (Figure 1C). SREBP itself also dimerizes and its dimerization is important for binding to FBXW7 (Osborne, 2000; Welcker et al., 2013).

#### 2.1.4 APC/C

**FIGURE 4:**
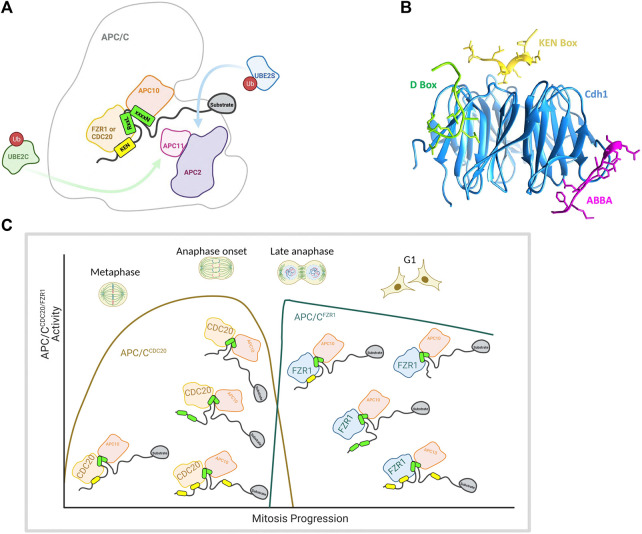
**(A)** Illustration of the cross section of the APC/C showing key subunits including the coactivator subunit (CDC20/FZR1), D-box co-receptor APC10, ubiquitin-bound E2s (UBE2C and UBE2S), catalytic subunits (APC2 and APC11) and a bound model substrate containing a KEN box and a D-box. **(B)** Structure of the three degrons (KEN (yellow), D-box (green) and ABBA (pink)) from APC/C-Cdh1 modulator 1 (Acm1) bound to *S. cerevisiae* APC/C activator protein Cdh1 (blue). The model was made in Chimera (https://www.rbvi.ucsf.edu/chimera) using PDB:4BH6 (He et al., 2013). **(C)** Schematic showing key APC/C subunits including coactivator subunit, E2 binding positions, and a bound model multivalent substrate containing a KEN box and a D-box Schematic showing proposed changes in substrate valencies during mitotic exit. D-boxes indicated in green, KEN motifs in yellow, Schematics were created with BioRender.com.

The above examples all illustrate that the presence of “repeated” degron motifs within a substrate (i.e., multiple degrons that all bind to the same site on the E3) allows for the possibility of cooperative binding to dimeric (or oligomeric) E3 subunits to promote substrate ubiquitination and degradation. APC/C presents an unusual case in that—as far as we know—it is the only E3 proposed to achieve multivalent interactions with substrates bearing multiple different degron motifs that are recognized by different binding sites on the substrate recognition subunit (Figure 1D). These degrons include the D-box, ABBA motif and KEN box (Davey and Morgan, 2016) that interact with the APC/C on docking sites created by the sub-stochiometric WD40-repeat protein substrate adaptor/co-activator subunits Cdc20 and Cdh1/FZR1 (FZR1) (Visintin et al., 1997). The existence of alternative substrate adaptors may contribute to ordering of substrate degradation by APC/C through differential degron preferences (Hartooni et al., 2022), notwithstanding the fact that the docking sites for D-box and KEN are highly conserved between Cdc20 and FZR1 and that in models of APC/C-substrate interaction, D-boxes and KEN motifs simultaneously occupy their docking sites (Barford, 2020) (Figures 4A,B). A cryo-EM study of the structure of APC/C^Cdh1/FZR1^ (APC/C^FZR1^) in complex with its pseudo-substrate inhibitor Acm1 revealed simultaneous engagement of D-box, KEN and ABBA motifs of Acm1 with their respective receptor sites on the co-activator, as evidence of co-operative binding of multiple degrons in APC/C interactions (Figure 4B) (He et al., 2013).

### 2.2 Allovalency

In contrast to the above examples, some E3-substrate interactions have been shown to require multiple substrate degrons even where there is only a single binding site on the E3 (Figure 1B). This is an example of allovalency, an extension of multivalency wherein a single binding site on the receptor (the E3) can bind to several identical epitopes on the ligand (the substrate). Allovalency enhances the E3-substrate interaction as when a bound substrate is released, the probability of rebinding is higher than expected based on substrate concentration alone because of the very high epitope concentration close to the binding site on the E3.

#### 2.2.1 Sic1/Cdc4

**FIGURE 5:**
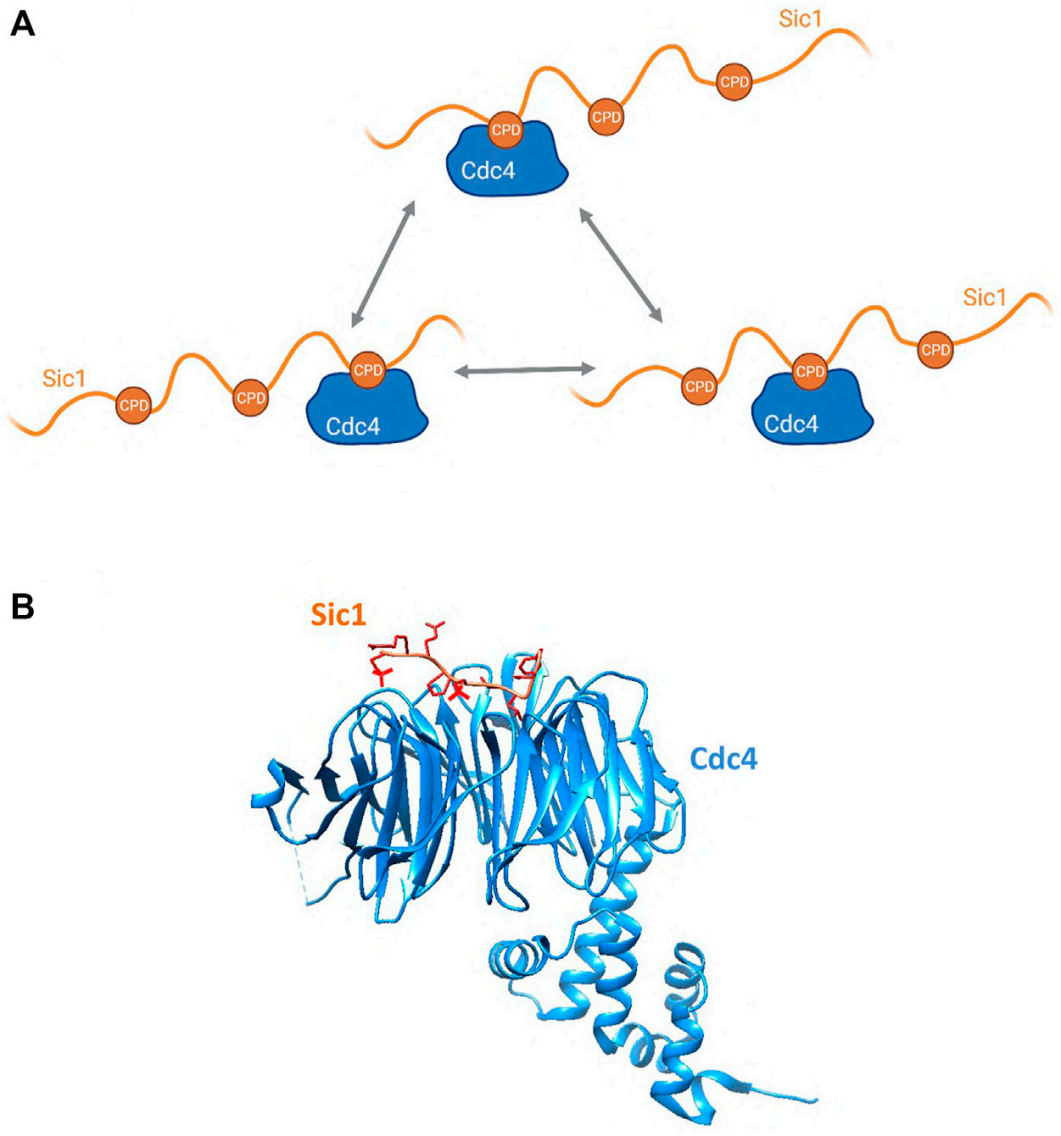
**(A)** Schematic showing the allovalent mechanism of interaction between the multiple phosphodegrons of the intrinsically disordered Sic1 and the E3 Cdc4, described in Section 2.2.1. Created with BioRender.com. **(B)** Structure of a Sic1 phosphodegron bound to Cdc4. Cdc4 is in blue and Sic1 in tan, with the phosphate groups shown in red. The model was made in Chimera (https://www.rbvi.ucsf.edu/chimera) using PDB:3V7D (Tang et al., 2012).

The defining example of allovalency is Sic1 and its interaction with Cdc4. Sic1 is a Clb5-Cdk1 inhibitor in *S. cerevisiae* that prevents premature S-phase entry (Schwob et al., 1994). For progression to S phase to occur, Sic1 must be degraded, and this is achieved through SCF^Cdc4^-mediated ubiquitination. Cdc4 is the substrate recognition subunit of the SCF ubiquitin ligase and contains a WD40 repeat domain through which it binds to Cdc4 phospho-degrons (CPD) on substrates (Figure 5). In Cdc4 substrates, phosphorylation of the CPDs is rapid and processive starting with Cln2-Cdk1 phosphorylation of T5, T33, T45 and S76 which then serve as docking sites for further phosphorylation by Clb5-Cdk1 (Kõivomägi et al., 2011). Several studies characterizing Sic1/Cdc4 binding have led to the identification of three multivalent modalities of the substrate-E3 interaction, namely allovalency (Figure 1B), E3 dimerization (Figure 1C), and allosteric recognition. First, Sic1 is intrinsically disordered and contains at least nine CPDs, all of which are low affinity degrons. No single degron alone is sufficient for Sic1 recognition by Cdc4—at least six CPDs are necessary—thereby making the interaction allovalent in character (Orlicky et al., 2003) (Figure 5). Second, *in vitro* studies have revealed that Cdc4 can form dimers and that Cdc4 dimerization is important for robust ubiquitination of Sic1 (Hao et al., 2007). Third, NMR perturbation studies have identified that Sic1 engages with an allosteric site on Cdc4 in a manner that would best fit with a negative allosteric interaction model rather than cooperative binding (Csizmok et al., 2017), and this interpretation is concordant with the allovalent model. The multiple CPDs on Sic1 engage with Cdc4 in dynamic equilibrium (Nash et al., 2001; Orlicky et al., 2003; Mittag et al., 2008), thereby establishing a threshold for Sic1 ubiquitination and degradation, and consequently S phase entry. This serves as a buffer mechanism to prevent premature cell cycle progression that could arise from possible fluctuations in Cdk activity (Nash et al., 2001; Orlicky et al., 2003; Mittag et al., 2010; Tang et al., 2012).

#### 2.2.2 Gli/β-TRCP

The Gli family of transcription factors are important players in the Hedgehog signaling pathway and can serve as activators or repressors based on their processed state. In the absence of Hh ligand, the transcription factors are processed into truncated repressor forms via ubiquitination mediated by SCF with F-box protein β-TrCP (SCF^βTRCP^) (Tempé et al., 2006). Phosphorylation of Gli1/2/3 on as many as 19 sites by a cascade of kinases including PKA, GSK3, CK1 and AMPK stimulates Gli processing by β-TrCP (Tempé et al., 2006; Wang and Li, 2006; Zhang et al., 2017; Shafique and Rashid, 2019). Gli3 lacks a consensus β-TrCP degron (DpSG [X_2-4_]pS), and binds β-TrCP via at least four non-canonical degrons (Tempé et al., 2006). This interaction is likely of an allovalent nature (Figure 1B), as β-TrCP contains only one degron binding site (Shafique and Rashid, 2019).

## 3 Multi-Degron Recognition by the APC/C

### 3.1 Degron Binding to the APC/C

Progression of cells out of mitosis involves ordered ubiquitin-mediated destruction of at least 100 different protein targets under control of the multi-subunit APC/C bound to one of its two coactivators (Min et al., 2014; Davey and Morgan, 2016). APC/C^Cdc20^ is a key component of the cell cycle machinery, with full activation of APC/C^Cdc20^ acting as the trigger for mitotic exit through targeted degradation of mitotic cyclins and securin (PTTG1) (Meadows and Millar, 2015). Coordination of mitotic exit events with segregation of duplicated chromosomes requires careful control of APC/C^Cdc20^ activity, which is achieved via the mitotic checkpoint that inhibits APC/C in the presence of faulty chromosome attachments to the mitotic spindle (Pines, 2011; Hein and Nilsson, 2014; Di Fiore et al., 2016; Yamaguchi et al., 2016; Alfieri et al., 2017; Watson et al., 2019). The mitotic checkpoint complex (MCC) prevents targeting of its critical metaphase substrates, whilst allowing degradation of a small number of so-called “checkpoint-independent” substrates such as cyclin A2 and Nek2A (Geley et al., 2001; Hayes et al., 2006). As cells exit mitosis, APC/C^Cdc20^ activity declines and is replaced with APC/C^FZR1^, which maintains activity until the end of G1 phase.

In the past 15 years, high resolution cryo-EM (electron microscopy) studies of APC/C structure, co-activator and substrate binding and recruitment of E2s has generated a richly detailed description of the structure-function relationships that govern degradation of its substrates (Barford, 2020). The binding of Cdc20 or FZR1 to the core APC/C creates at least three degron binding pockets (receptors) for the known APC/C degron SLiMs, namely the widespread “Destruction-box” (D-box, consensus RxxLxxxxN) and KEN motifs, and the more restricted ABBA motif thought to be required for Cyclin A degradation only, and not further discussed in this review (Figure 4B). The KEN motif docks to the top surface of the WD40 propeller of the co-activator and the D-box to a cleft formed between two blades of the propeller and the neighbouring APC10 subunit such that substrate engagement with degron receptors is likely to stabilize the active complex (Burton et al., 2005; Matyskiela and Morgan, 2009; Buschhorn et al., 2011; da Fonseca et al., 2011; Chang et al., 2014). This feature may explain the broad variation in D-box sequences (and loose consensus thereof): whereas the critical residue of the D-box, leucine at position 4 (P4) contacts a hydrophobic pocket in the co-activator subunit, the ‘tail’ of the D-box degron and its flanking sequence (P8-12) contact the APC10 subunit and in doing so may influence the efficiency of substrate ubiquitination (Chang et al., 2014; Qin et al., 2019). The lack of conservation at these positions in D-box sequences would therefore be consistent with differential efficiency of ubiquitination of substrates with variant D-boxes.

In conclusion, it seems likely that individual degrons influence the degradation rate of their substrates through mechanisms independent of their affinities (Davey and Morgan, 2016). Nonetheless the idea that the affinity of APC/C-substrate interactions is crucial to understanding how the APC/C “orders” the degradation of its substrates remains central. Recent reviews of this question have discussed proposed mechanisms that include co-activator switching, fine-tuning of APC/C-substrate interactions by phosphorylation or other post-translational modifications, differential processivity of ubiquitination, and substrate competition (Davey and Morgan, 2016; Alfieri et al., 2017; Bodrug et al., 2021).

### 3.2 Multivalent Binding

There is little known about how substrates compete with each other and how multivalency (both in the sense of existence of different degrons and of multiple copies of the same degron) act as mechanisms to confer specificity and affinity to their interactions with the APC/C. Recent high-resolution structural studies of the APC/C in complex with inhibitors and substrates simultaneously occupying multiple binding sites have led to the expectation that cooperative binding of multiple degrons may be required for productive target engagement. However the crystal structure showing simultaneous occupancy of KEN, D-box and ABBA receptor sites on FZR1 uses Acm1, a pseudo-substrate inhibitor that may have distinct binding characteristics not shared by substrates (He et al., 2013). Indeed, pseudo-substrates should bind more tightly than the substrates with which they compete, and a recent study has shown that mutating one of the D-boxes in Acm1 turns it into a degradable substrate of APC/C (Qin et al., 2019). An interesting recent cryo-EM study of the substrate cyclin A2 bound to APC/C^Cdc20^ reveals alternative substrate poses mediated by two D-boxes, D1 and D2 (Zhang et al., 2019). In the presence of the inhibitory mitotic checkpoint complex (MCC), all cyclin A2 degrons are engaged, with the KEN box bound to Cdc20^APC/C^, the ABBA motif to Cdc20^MCC^, and D2 bound to the D-box Receptors (DBR) present on Cdc20^APC/C^ and Cdc20^MCC^ respectively (Alfieri et al., 2016; Yamaguchi et al., 2016; Barford, 2020). The other D-box, D1, contributes to avidity and ensures that cyclin A2 can be ubiquitinated by the APC/C in presence of MCC. In the absence of MCC, the DBR on Cdc20^APC/C^ is shown to engage with either D1 or D2, but always with simultaneous binding of KEN. Although the binding of cyclin A2 represents a ‘special case’ in substrate terms, it appears to confirm both simultaneous engagement of KEN receptor, ABBA receptor and DBRs by a substrate, which favours a co-operative binding model and the possibility of alternative engagement of the same degron.

A recent and highly informative study of binding kinetics of degron peptides to immobilised APC/C complexes (Visintin et al., 1997; Hartooni et al., 2022) clearly demonstrates cooperativity of binding of KEN and D-box degrons with estimated 100-fold increase in affinity conferred by the addition of a KEN motif to a D-box-only peptide. However, despite compelling evidence for cooperative multivalent interactions of substrate with APC/C via distinct degrons, the observation of the presence of multiple repeats of degrons of the same type within many substrate sequences (see Table 1) remains unexplained. This calls into question the model that cooperative binding alone drives the affinity for APC/C conferred by multiple degrons and raises the possibility that additional degron sequences contribute to robust degradation through allovalent effects. Indeed, the striking enrichment for degrons (and KEN motifs in particular) in known APC/C substrates indicates that they are likely functional motifs (Liu et al., 2012; Min et al., 2014).

**TABLE 1:** Contains information on degrons in well-known APC/C substrates, scored according to the ProVIZ SLI degron tool http://slim.icr.ac.uk/apc/index.php.

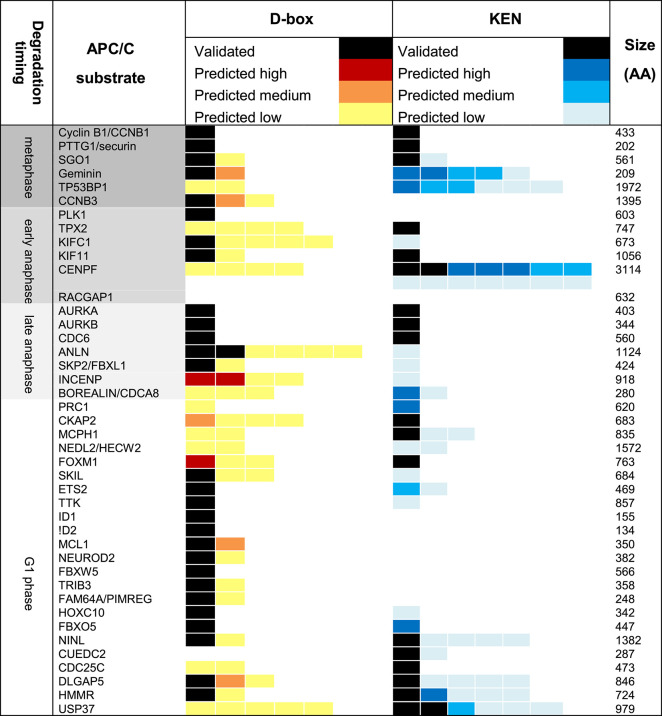

Only substrates targeted at mitotic exit are included. Our study does not include APC/C substrates degraded whilst the SAC is active (such as cyclin A, Nek2A, and KIF18), shown to have additional D-box- and KEN-independent docking sites on APC/C. For each substrate, degrons validated in the literature are included (black tabs), alongside degrons predicted by ProViz (one tab per degron). Predicted degrons are included where disorder score ≥0.4 and similarity to consensus motif scores >0.5 (low), >0.8 (medium) or >0.85 (high).

## 4 Substrate Ordering in Mitosis: Multivalency or Allovalency?

The longstanding question of how temporal ordering of degradation of APC/C substrates is achieved remains a perplexing one. Although a number of mechanisms have been shown to contribute to timing of degradation of individual substrates (Bansal and Tiwari, 2019), few generalities have been extracted so far. For example, phosphorylation of degrons can block or enhance substrate degradation, so the progressive dephosphorylation of substrates (and APC/C) from metaphase onwards could contribute to a changing landscape of APC/C-substrate interaction. Substrate-specific context beyond the D-box remains poorly understood. For example, the identity of ubiquitin acceptor lysines in most degradative ubiquitination reactions is unknown. This is likely a critical determinant of the efficiency of substrate degradation through “processive affinity amplification”, a feed forward effect whereby ubiquitinated substrates show increased affinity of APC/C binding relative to the unmodified substrate (Lu Y. et al., 2015; Brown et al., 2016).

Notwithstanding, competition between substrates for binding to the APC/C is an intuitive explanation that re-defines the question as one of self-ordering of substrates. As proposed in an early study of this question (Rape et al., 2006), substrates with greater affinity for the APC/C would undergo the most “processive” ubiquitination (i.e., the addition of multiple ubiquitin moieties in a single binding event) and so would be degraded earlier, at the expense of substrates with lower affinity. We now know that in the case of two well studied examples—cyclin A before cyclin B and Plk1 before AURKA—the timing of degradation in cells is set by specific mechanisms (inactivation of SAC and activation of FZR1, respectively) (Geley et al., 2001; Lindon and Pines, 2004; van Zon and Wolthuis, 2010). However, for the vast majority of APC/C substrates, self-ordering through substrate degron competition is predicted to contribute to the rate and timing of degradation (Lu D. et al., 2015).

Would differences in affinity substrate competition arise just from the quality of single degrons, or from their quantity—that is, what are the contributions of multivalency and allovalency to substrate ordering? To investigate further the role of degron “quantity”, we used the bioinformatic resources created by Norman Davey and colleagues (http://slim.icr.ac.uk/apc/) to map the incidence of validated and predicted degrons in a panel of known substrates of the APC/C (Table 1). We excluded from our Table the pseudo-substrate inhibitors of the APC/C (BUB3, Acm1 EMI1) and the substrates degraded when MCC is active (Cyclin A, Nek2, and KIF18A) since these are thought to bind the APC/C via additional routes (multivalent degrons, IR/LR tail, and Cks1) (Di Fiore and Pines, 2010; Sedgwick et al., 2013) and included substrates degraded during mitotic exit (that is, from metaphase onwards). Although there are limited data available on validated degrons and timing of substrate degradation beyond a few key examples, by combining the available information with degron predictive algorithms, we propose the following generalizations (Figure 4C):

### 4.1 Metaphase: The Active APC/C

The metaphase substrates Cyclin B1 and securin/PTTG1 possess strictly one D-box and one KEN motif, both essential for degradation, indicating that simultaneous, cooperative binding to DBR and KEN receptor sites on APC/C underpin the most critical function of the APC/C (as in Figure 1D). The highly conserved and identical modality of binding shared by Cyclin B1 and securin/PTTG1 is notable in light of the elegant study from the Hauf group showing how competition between these substrates for APC/C activity modulates their respective degradation rates to buffer downstream events against fluctuations in protein level, thus maintaining robust ordering of anaphase events (Kamenz et al., 2015). Other substrates known to be degraded during metaphase show variable degron numbers. The predictions for multiple copies of either D-box or KEN motif points towards allovalency as a mechanism to promote interaction of these “secondary” substrates in the presence of Cyclin B1 and securin/PTTG1. We note that onset of both geminin and cyclin B3 degradation appears later than that of cyclin B1 (Nguyen et al., 2002; Clijsters et al., 2013), which would be consistent with the idea that these other metaphase substrates (relying on monovalent or allovalent receptor-degron interactions, Figures 1A,B) do not compete efficiently with the multivalent interactions of Cyclin B1 and securin/PTTG.

### 4.2 Anaphase Onset: The Second Wave of Peak APC/C Activity

Anaphase onset coincides with a new wave of APC/C^Cdc20^ activity that appears able to target substrates not degradable by metaphase APC/C^Cdc20^, including D-box-deleted versions of securin/PTTG1 and Nek2A (Hagting et al., 2002; Hayes et al., 2006). The relaxed specificity of APC/C^Cdc20^ does not have a mechanistic explanation but is assumed to result from a dephosphorylation cascade that drives mitotic exit (Hein et al., 2017). However, it is also possible that once the high affinity substrates (those exhibiting cooperative multivalency) have been cleared from the cell, the same APC/C activity in fact becomes free to target a new wave of substrates exhibiting a range of kinetic interactions, including non-cooperative ones. Indeed, some natural substrates targeted early in anaphase appear to rely on a single degron, or show low numbers of predicted degrons (Plk1, KIF11, and KIFC1) (Lindon and Pines, 2004; Min et al., 2014; Singh et al., 2014). The question of degron dependence could be addressed through experiments with the competitive D-box inhibitor APCin (Sackton et al., 2014), where substrates dependent on a single D-box for degradation would be more sensitive to the inhibitor than substrates exhibiting multivalent cooperative binding. In summary, we propose that the broadening of substrate specificity at anaphase onset reflects a relaxation of requirements for efficient substrate ubiquitination such that efficient ubiquitination of substrates can be achieved in the absence of cooperative engagement to DBR and KEN receptor (Figure 1D) and multivalency becomes dispensable.

### 4.3 Late Mitosis and G1: Fading APC/C Activity

Falling mitotic CDK activity during anaphase leads to dephosphorylation and activation of the APC/C co-activator FZR1, with onset of Aurora kinases degradation (AURKA and AURKB) the earliest known marker for active APC/C^FZR1^. From this time onwards, the presence of multiple degrons appears to be a condition of degradation of mitotic substrates, with two or more degrons required in substrates where degrons have been validated (Aurora kinases, CDC6) (Petersen et al., 2000; Littlepage and Ruderman, 2002; Crane et al., 2004; Mailand and Diffley, 2005). There are multiple predicted degrons in many other substrates (e.g., ANLN, CENPF) (Table 1) indicating a potential role for multiple copies of the same degron in driving allovalent effects (Figure 1B). A study of yeast Cdh1/FZR1 substrates concluded that the DBR was essential for degradation of all substrates, even those lacking a cognate D-box, but that the KEN receptor was essential for degradation of only a subset of substrates (Qin et al., 2016). For substrates where KEN receptor is dispensable it may be that alternative interactions (for example mediated through ABBA receptor) or allovalency effects at the D-box receptor compensate for D-box/KEN cooperativity in driving substrate-E3 interactions.

The degradation of APC/C substrates continues through mitotic exit into G1 phase, when APC/C activity becomes exclusively dependent on FZR1, and where many new substrates appear to reply on a single degron. We propose that multivalency is important for efficient targeting of anaphase substrates by FZR1, allowing them to outcompete other substrates, but that reduced competition in G1 phase renders multi- or allo-valency of degrons dispensable.

## 5 Perspective

The well-debated issue of substrate ordering by the APC/C is usually expressed, in the cell cycle field, along the lines of “how does the APC/C recognize so many substrates with such exquisite specificity of timing?”, posing the question as one to be solved by unravelling the complexity of the APC/C. With new perspectives on substrate targeting that have come into view through the lens of TPD strategies, we have instead looked for an answer to the converse question: ‘how do so many substrates access the activity of the APC/C to bring about their degradation at the right time?’ Answers to this question will inform new strategies for harnessing the APC/C, and other E3s, in the design of TPD tools.

We first considered examples from the scientific literature where multivalency of degron motifs is known to play a role in determining degradation of a substrate, to review the roles of multivalency in influencing substrate-E3 interactions (summarized in Figure 1). We then looked more closely at available information on APC/C substrates, concluding that there is a role for both multiple types of degrons to generate cooperativity, and multiple repeats of degrons to enhance affinity of interactions. Whereas the distribution of degrons in metaphase substrates is consistent with a role for distinct D-box and KEN degrons binding cooperatively to optimize interaction with APC/C^Cdc20^, this may not be the case throughout mitosis. The variability in degron numbers, with some substrates carrying multiple copies of predicted degrons, argue in favour of multivalency as a mechanism for enhancing affinity, perhaps to generate threshold effects to determine the order of substrate degradation.

Multivalency could thus be viewed as a useful tool for enhancing substrate affinity for an E3 ligase in the design of TPD tools. Indeed, the use of a “trivalent” PROTAC in a different configuration (two ligands for cooperative binding to the target substrate and the third to recruit the E3) was recently reported (Imaide et al., 2021). Multiple degrons could be used also to enhance recruitment of E3s, even where there is a single copy of the degron receptor present (as is the case for the APC/C). Of particular interest for the design of tools to harness APC/C, we note that multimerizing single degrons to create allovalency could be a way of promoting affinity of a neo-substrate for the APC/C without competing with the critical metaphase substrates that exhibit co-operative multivalent binding through D-box and KEN. Such neo-substrates might be expected to degrade later during mitotic exit or G1 phase.

The variety of APC/C degron sequences, which has hindered attempts to establish the rules of engagement of APC/C with its substrates, is undoubtedly a feature of the variation in timing of substrate degradation that we are attempting to understand. The evolution of degrons within substrates allows fine-tuning of their binding to APC/C and efficiency of resulting ubiquitination, and a key element of this process is the existence of multiple degrons embedded as SLiMs in rapidly evolving IDRs. Therefore, degron repeats, degron shuffling and degron spacing, as well as degron sequences themselves, can all be evolving features that substrates use to compete with each other for access to the APC/C and/or to modulate the conformation and activity of the APC/C.

Thus, substrates evolve to control the efficiency of their own ubiquitination, and a better understanding of this process can contribute to the design of new biological tools that dock neo-substrates onto cellular E3s for therapeutic purposes.
